# Supplementary material for: Observing polarization patterns in the collective motion of nanomechanical arrays
Source: Nat Commun. 2022 May 5;13:2478. doi: 10.1038/s41467-022-30024-0 (PMC9072344; doi:10.1038/s41467-022-30024-0)
Supplement: Supplementary file 1 — Supplementary Information [file 41467_2022_30024_MOESM1_ESM.pdf]

Supplementary Information: Observing polarization patterns in the collective motion of nanomechanical arrays

Juliane Doster,[1,*] Tirth Shah,[2,3,*] Thomas Fösel,[2,3] Philipp Paulitschke,[4] Florian Marquardt,[2,3] and Eva M. Weig[1,5,6,†]

[1] *University of Konstanz, Department of Physics, Universitätsstr. 10, 78457 Konstanz, Germany*
[2] *Max Planck Institute for the Science of Light, Staudtstr. 2, 91058 Erlangen, Germany*
[3] *Friedrich-Alexander University Erlangen-Nürnberg (FAU), Department of Physics, Staudtstr. 7, 91058 Erlangen, Germany*
[4] *Ludwig-Maximilians-Universität Munich, Department of Physics, Geschwister-Scholl-Platz 1, 80539 München, Germany*
[5] *Technical University of Munich, Department of Electrical and Computer Engineering, Hans-Piloty-Str. 1, 85748 Garching, Germany*
[6] *Munich Center for Quantum Science and Technology (MCQST), Schellingstr. 4, 80799 München, Germany*

(Dated: April 26, 2022)

Supplementary Note 1. IMAGING SETUP

We employ optical detection by means of microscopy to simultaneously detect the dynamics of every nanopillar within the array. The array is imaged from above, which allows to capture the envelope of each pillar's trajectory, resolving not only its amplitude but also the vibration direction, which gives access to the polarization degree of freedom. This measurement technique is enabled by the relative large vibrational amplitudes of nanomechanical pillar resonators even in the linear response regime.

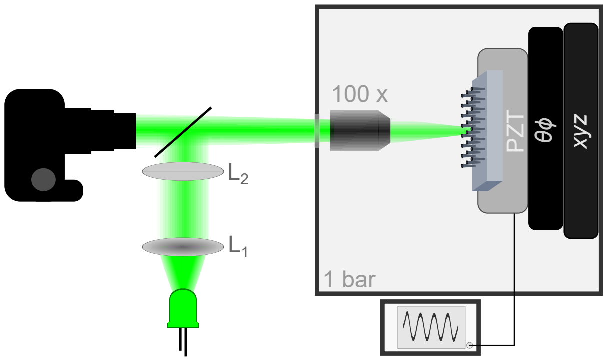

Supplementary Figure 1. Schematic of the imaging setup for the detection of nanomechanical pillar arrays. Light from a green LED is diffused via a lens L1 exhibiting a grained surface and collimated via a second lens L2. The light is then focused onto the sample via a 100× objective. The light reflected off the sample passes through a beamsplitter and is captured in a DSLR camera. The sample is driven via a piezoelectric transducer (PZT), that is connected to a signal generator. A combination of a xyz- and a $\theta\phi$-stage are employed for precise sample positioning.

A schematic drawing of the experimental setup is shown in Supplementary Figure 1. The imaging detection system consists of a home-built microscope operating with monochromatic light to avoid imaging distortions from chromatic abberation. The light of a green LED is focused on the pillar array from the top through a 100× magnification objective. A diffuser lens with a grained surface (L1) is employed to homogenize the illuminating light across the large field of view (approx. $100 \times 100\,\mu\text{m}^2$) required to capture the pillar array. In addition, 2 inch optic components are employed to ensure the uniform illumination of the array. The light reflected from the sample is then captured via a digital single-lens reflex (DSLR) camera. Green light is chosen to exploit the spatial sensitivity maximum of the camera chip.

---

* These two authors contributed equally to this work
† eva.weig@tum.de

The sample is positioned with a remote controlled xyz-stage and a mechanical $\theta\phi$-tilt-stage. In particular, precise tilt correction is required to tune the entire field of view into focus. This is crucial to discriminate both idle and vibrating pillars, the envelopes of which appear as blurred circles and shapes (see Figures 1**c-e** of main text), against unfocussed ones. To compensate for position or focus drifts, that might influence the interpretation of the images, reference images with the drive switched off are taken before every image capture of the driven sample.

The nanopillars of the array are driven via a shear piezoelectric transducer glued underneath the sample chip. As the single image capture time of the camera is approx. 1 s and thus much larger than the oscillation period of the pillars at eigenfrequencies of about 1 MHz, the trajectories are not time-resolved but rather the integrated trajectory is obtained, yielding an image of the resulting envelopes.

All measurements in this article are taken at room temperature and atmospheric pressure.

Supplementary Note 2. EXTRACTING MOTION FROM THE BLURRED PILLAR IMAGE

In this section, we outline the image processing algorithm that is used to extract the pillar trajectory from the stationary and moving pillar images. The underlying idea behind the algorithm is presented in Figure 1**f** of the main text. It states that the moving pillar image is reconstructed as the convolution of the stationary pillar image with the probability distribution of the position along the fitted elliptical trajectory (taking into account the time spent inside each interval along the trajectory). The fitting procedure works by optimizing the ellipse trajectory parameters to match the reconstructed moving pillar image with the experimentally observed one.

Our goal is to determine the parameters $\boldsymbol{\lambda} = (x_0, y_0, A\cos\zeta, A\sin\zeta, \theta)$ of the elliptical trajectory, where (x_0, y_0) is the center position of the ellipse, $\{A\cos\zeta, A\sin\zeta\}$ are the lengths of its two main axes, and θ is the orientation of the

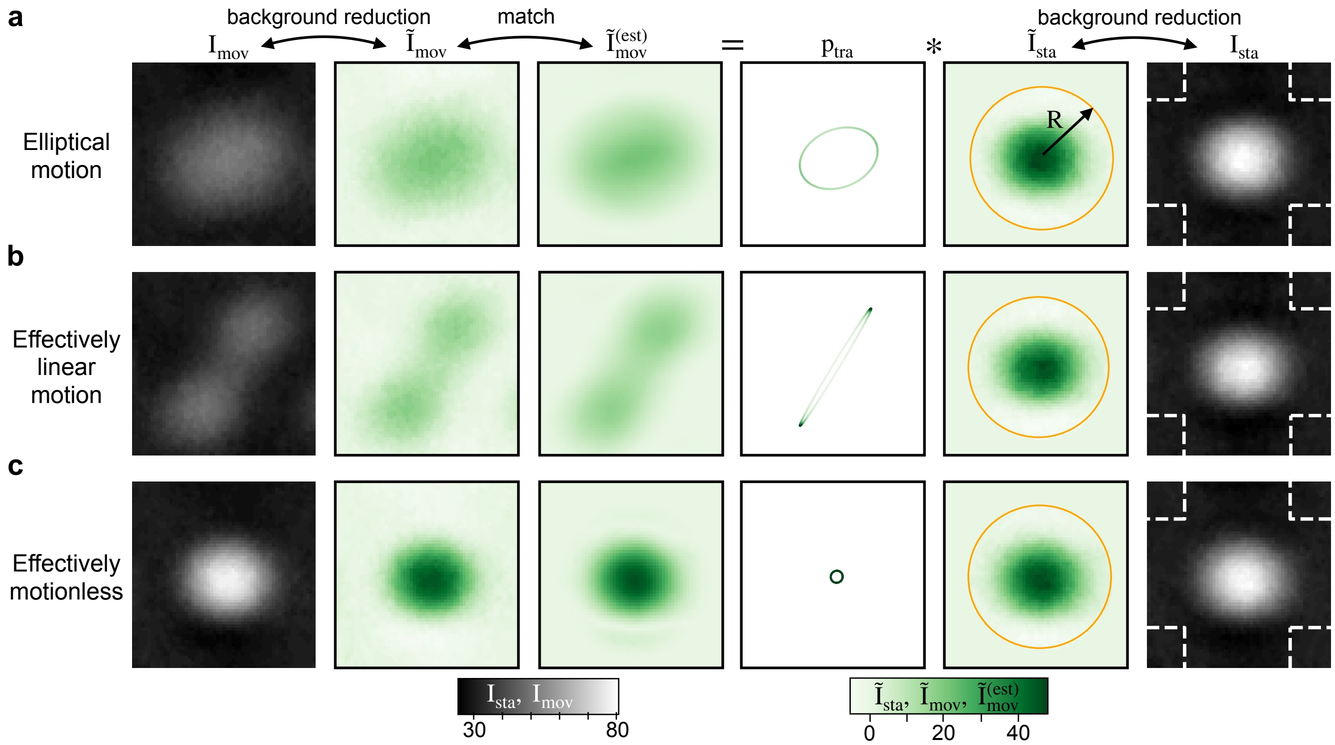

Supplementary Figure 2. Image processing algorithm to extract pillar trajectories from the blurred pillar images. Demonstration of the algorithm with qualitatively different trajectories: **a**, elliptical motion, **b**, effectively linear motion, and **c**, effectively motionless. The first (last) two columns show the pixel intensity of the moving $I_{\rm mov}$ (stationary $I_{\rm sta}$) pillar and the corresponding background reduced intensity distribution $\tilde{I}_{\rm mov}$ ($\tilde{I}_{\rm sta}$); subsequent three columns depict the convolution equation $\tilde{I}^{\rm (est)}_{\rm mov} = \tilde{I}_{\rm sta} * p_{\rm tra}$; the estimated distribution $\tilde{I}^{\rm (est)}_{\rm mov}$ is compared with the moving pillar distribution $\tilde{I}_{\rm mov}$ to yield the overlaps 0.992, 0.969, 0.999 for the three cases (a-c). The background distribution $I_{\rm bg}$, described in Step 1 of the algorithm, is obtained by interpolating the mean intensities near the four corners of the static pillar image (indicated with dashed lines in the last column) to the complete 2D cropped image.

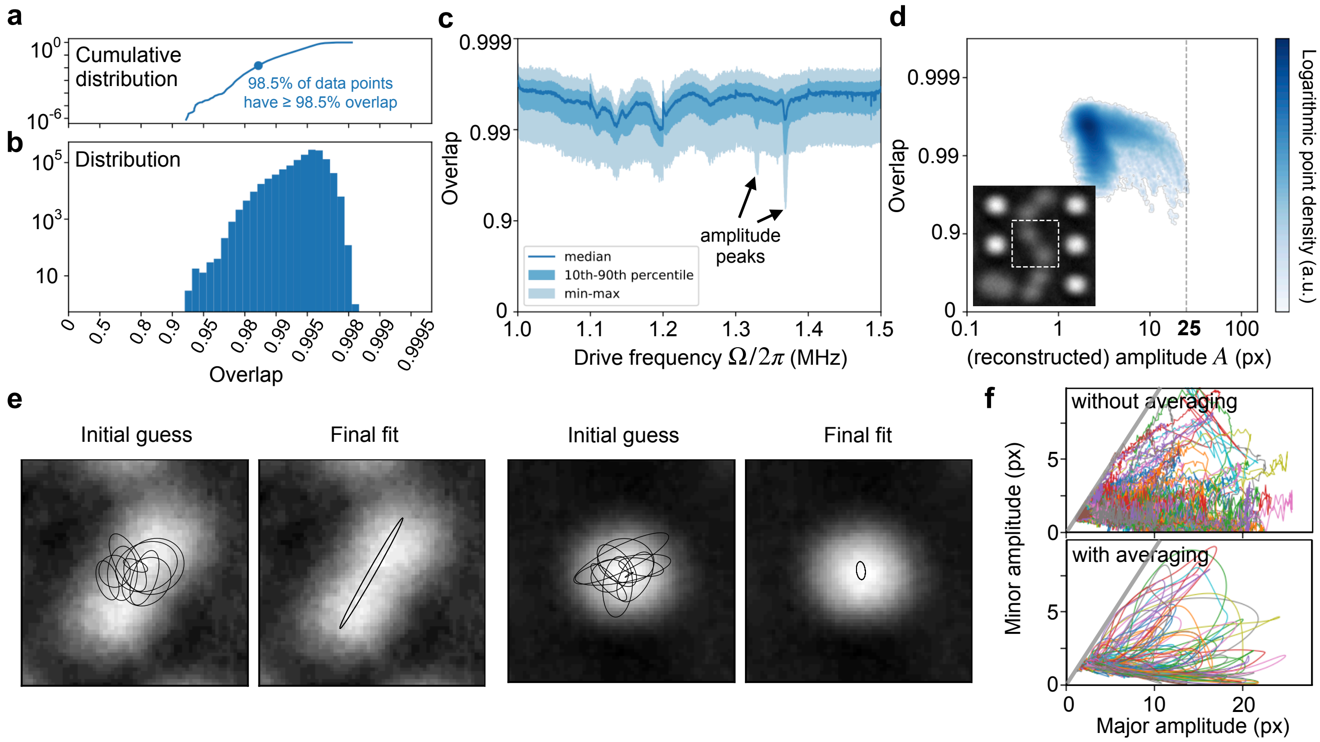

Supplementary Figure 3. **Characterizing the performance of the image analysis algorithm.** **a-b**, Cumulative distribution and histogram of the overlap O over the central 16×16 pillars and all driving frequencies Ω. **c**, Overlap O as a function of the driving frequency Ω. It features dips at the frequencies of maximum amplitude response. The large difference between the median (dark blue) and the minimum value (light blue) at the two largest amplitude peaks indicates that only a minor fraction of the pillars have smaller overlaps. **d**, Overlap O as a function of the reconstructed amplitude A. The overlap decreases with larger amplitude, thereby explaining the dips in (**c**). For the large amplitudes of approximately half the unit-cell size (dashed vertical line), the overlap gets worse because either the analyzed pillar trajectory or its neighboring pillar trajectories can cross the cropping region (the inset shows such an example with cropping region of the central pillar indicated in white). **e**, Demonstration of the robustness of the optimization process for two different pillar trajectories. A large number of the initial condition of the trajectories converges to only one estimated trajectory after the optimization. **f**, Frequency evolution of the estimated pillar trajectories as a major vs. minor amplitude plot (Figure 4 of the main text) with (bottom) and without (top) the Gaussian filter averaging over neighboring frequencies.

major axis (cf. Figure 2**a** of the main text):

$$\mathbf{r}(t) = \begin{pmatrix} x_0 \\ y_0 \end{pmatrix} + AR(\theta) \begin{pmatrix} \cos\zeta \cos(\Omega t) \\ \sin\zeta \sin(\Omega t) \end{pmatrix}. \tag{1}$$

Here, $R(\theta)$ is the rotation matrix given by

$$R(\theta) = \begin{pmatrix} \cos\theta & -\sin\theta \\ \sin\theta & \cos\theta \end{pmatrix}. \tag{2}$$

For this purpose, we apply the following steps:

1. From the raw image of a static (moving) pillar, we obtain the gray-scale pixel intensity distribution $I_{\mathrm{sta(mov)}}(\mathbf{r})$. This distribution is defined within a fixed cropping region (here, $1.2 \times$ unit-cell size) centered at the rest position of the pillar, cf. Supplementary Figure 2. It would be inaccurate to perform the convolution operation directly on the raw distribution $I_{\mathrm{sta}}(\mathbf{r})$ to compare with the $I_{\mathrm{mov}}(\mathbf{r})$. This is because: (i) the intensity near the boundaries of the cropped static pillar image is non-zero (the region near the boundaries are static, so they should not be convolved and hence should be zero), (ii) there is a gradient in the intensity along the cropped image (e.g. one side of the cropped image is brighter than the other). For these reasons, we obtain the processed static (moving) pillar intensity distribution $\tilde{I}_{\mathrm{sta(mov)}}(\mathbf{r})$ by subtracting the background distribution $I_{\mathrm{bg}}(\mathbf{r})$ from the raw static (moving) distribution $I_{\mathrm{sta(mov)}}(\mathbf{r})$. The background distribution $I_{\mathrm{bg}}(\mathbf{r})$ is obtained via a bilinear interpolation

of the mean pixel intensities at the four corners of the static pillar image to the complete 2D cropping region. Furthermore, to ensure that the distribution $\tilde{I}_{\rm sta}(\mathbf{r})$ is exactly zero away from the pillar, we clip it to zero for distance greater than a fixed R from the center. Thus, the processed static and moving pillar distributions are given by

$$\tilde{I}_{\rm sta}(\mathbf{r}) = \left(I_{\rm sta}(\mathbf{r}) - I_{\rm bg}(\mathbf{r})\right)\Theta(R - |\mathbf{r}|), \quad \tilde{I}_{\rm mov}(\mathbf{r}) = I_{\rm mov}(\mathbf{r}) - I_{\rm bg}(\mathbf{r}). \tag{3}$$

Here, $\Theta(x)$ is the Heaviside step function. Note that the distribution functions $\tilde{I}_{\rm sta(mov)}(\mathbf{r})$ can also take negative values. Moreover, the pixel intensity gradient effects are extremely substantial near the edges of array, where we cannot reliably extract the pillar trajectories. Therefore, we perform the fit only over the central 16×16 pillars rather than the full array comprising of 20×20 pillars.

2. The probability distribution of the elliptical trajectory (accounting for the time spent along different intervals of the trajectory) is written as

$$p_{\rm tra}(\mathbf{r}) = \frac{\Omega}{2\pi}\int_0^{2\pi/\Omega} \delta(\mathbf{r} - \mathbf{r}(t))\,\mathrm{d}t \tag{4}$$

For the optimization, the center coordinates x_0 and y_0 are initialized via the mean position, whereas the other parameters are initialized via the covariance matrix, cf. Supplementary Note 4 (mean and variances are evaluated using the moving pillar distribution $\tilde{I}_{\rm mov}(\mathbf{r})$).

3. Next, we estimate the distribution of the moving pillar image $\tilde{I}^{\rm (est)}_{\rm mov}[\boldsymbol{\lambda}](\mathbf{r})$ via the convolution relation

$$\tilde{I}^{\rm (est)}_{\rm mov}[\boldsymbol{\lambda}](\mathbf{r}) = (\tilde{I}_{\rm sta} * p_{\rm t})(\mathbf{r}) = \int d\mathbf{r}'\ \tilde{I}_{\rm sta}(\mathbf{r} - \mathbf{r}')\ p_{\rm tra}[\boldsymbol{\lambda}](\mathbf{r}'). \tag{5}$$

The ellipse parameters $\boldsymbol{\lambda}$ are iteratively optimized to minimize the mean squared error

$$\int_{\mathbb{R}^2} [\tilde{I}^{\rm (est)}_{\rm mov}(\mathbf{r}) - \tilde{I}_{\rm mov}(\mathbf{r})]^2\,\mathrm{d}^2 r \tag{6}$$

We use gradient descent (Hessian method) for optimization, whose advantage is that it converges very rapidly.

The algorithm is demonstrated for the three qualitatively diverse test-cases in Supplementary Figure 2. In addition, the results of a detailed characterization of all the pillars (for all driving frequencies) by the algorithm are presented in Supplementary Figure 3, which can be summarized as follows:

(i) Image analysis performance for different amplitudes: We quantify the performance of the image analysis by investigating the optimized overlap function

$$O = \frac{\int d^2r\, \tilde{I}^{\rm (est)}_{\rm mov}(\mathbf{r})\tilde{I}_{\rm mov}(\mathbf{r})}{\sqrt{\int d^2r\, \left[\tilde{I}^{\rm (est)}_{\rm mov}(\mathbf{r})\right]^2 \int d^2r\, \left[\tilde{I}_{\rm mov}(\mathbf{r})\right]^2}} \tag{7}$$

in Supplementary Figure 3**a-d**. The distribution of the overlap function reveals that the reconstructed image is practically identical to the observed image for almost all the cases, cf. Supplementary Figure 3**a,b**. Additionally, we observe that the large amplitude pillars typically have smaller overlaps (see Supplementary Figure 3**c,d**). This is because at higher amplitudes, the pillar trajectories are very near to (or even outside) the boundary of the cropping region.

(ii) Robustness of the optimization: One can test the robustness of the algorithm by analyzing the final fit results of the optimization process for many different initial trajectories. In Supplementary Figure 3**e**, we see that all the initial guesses converge to only one final trajectory.

(iii) Improving the data reliability via averaging: A more direct way to test the reliability of the extracted pillar motion is to investigate the frequency evolution of a single pillar trajectory. As can be seen in Supplementary Figure 3**f**, the extracted trajectory of the pillar features strong fluctuations, which arises from the combined effects of the data acquisition and the image analysis. These fluctuations can be smoothed-out via a Gaussian averaging of the optimized ellipse parameters over neighboring frequencies.

Supplementary Note 3. THEORETICAL DESCRIPTION OF THE MOTION OF A SINGLE PILLAR

In Figure 2 of the main text, we describe the frequency response of a single pillar and observe its transition from linear polarization to elliptical and back to linear. In this section, we derive the equation of this trajectory, and explicitly illustrate that a non-zero mechanical damping Γ is necessary in order to observe elliptical trajectories.

Without any loss of generality, we assume that the two normal modes of the pillar are oriented along the x and y directions, with corresponding frequencies ω_x and ω_y. In terms of the complex displacement amplitude defined as $b_x = \sqrt{\omega_x/2}(x + i\dot{x}/\omega_x)$ and likewise for b_y, the Hamiltonian is given by

$$H = \omega_x b_x^* b_x + \omega_y b_y^* b_y. \tag{8}$$

Here, we did not consider any possible additional coupling term between x and y of the form $-J(b_x^* b_y + b_y^* b_x)$. This is because even if we would have included the coupling term, then one can still diagonalize the resulting Hamiltonian to obtain the Hamiltonian of two uncoupled harmonic oscillators in two orthogonal directions (other than x and y).

For an external harmonic drive at frequency Ω and orientation φ with respect to the x-axis, the equation of motion is written as

$$\frac{db_s}{dt} = -i\frac{\partial H}{\partial b_s^*} - \frac{\Gamma}{2}b_s + if_s e^{-i\Omega t}, \quad s = \{x, y\}. \tag{9}$$

Here, the partial derivative $\partial H/\partial b_s^*$ is taken only over b_s^*, while b_s is held as a constant. The force amplitude f_x (f_y) is proportional to $\cos\varphi$ ($\sin\varphi$). Thus, the steady state solution is evaluated to be

$$b_s = \frac{f_s e^{-i\Omega t}}{(\omega_s - \Omega) - i\Gamma/2}. \tag{10}$$

The shape of the elliptical trajectory can be obtained from this solution according to the procedure described in Supplementary Note 4.

We now analyze the polarization physics of this trajectory with respect to the mechanical damping Γ. The phase lag between the two polarizations (phase of b_y/b_x) determines the ellipticity. For a drive parked at the central frequency $\Omega = \omega \equiv (\omega_x + \omega_y)/2$, the phase lag is given by

$$b_y/b_x = \left(\frac{-1 - i\Gamma/(2\Delta)}{1 - i\Gamma/(2\Delta)}\right)\tan\varphi. \tag{11}$$

Here, $\Delta = (\omega_y - \omega_x)/2$ is the frequency anisotropy. Note that for the two extreme values of the mechanical damping $\Gamma/(2\Delta) \ll 1$ and $\Gamma/(2\Delta) \gg 1$, the phase of b_y/b_x converges to π and 0 respectively, indicating that the trajectory is mainly linear for these cases. For $\Gamma = 0$, the phase of b_y/b_x is exactly π. Hence, the elliptical trajectories can only be observed by breaking the time-reversal symmetry with finite dissipation. This can be illustrated by visualising the frequency evolution of the pillar trajectory on the Poincaré sphere for different values of Γ, cf. Supplementary Figure 4.

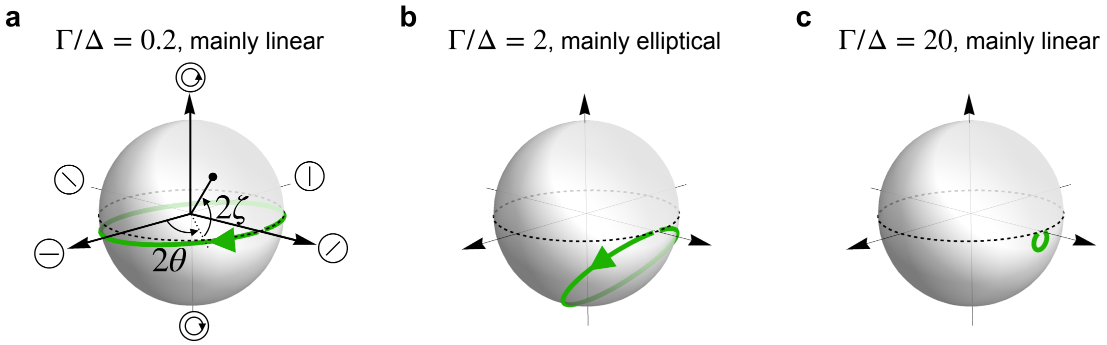

Supplementary Figure 4. **Polarization physics analysis of a single pillar with respect to mechanical damping.** Evolution of the pillar trajectory on the Poincaré sphere as a function of the drive frequency Ω (frequency increasing in the direction of green arrow) for three different values of the damping Γ relative to the frequency anisotropy Δ: **a**, $\Gamma/\Delta \ll 1$, **b**, $\Gamma/\Delta \approx 1$, **c**, $\Gamma/\Delta \gg 1$. The trajectory is majorly elliptical (passing close to the poles of the Poincaré sphere) only for intermediate values of the damping $\Gamma/\Delta \approx 1$. [Parameter values: $\omega_x = 0.95, \omega_y = 1.05, \varphi = 45°$.]

Supplementary Note 4. DETERMINING SHAPE OF THE ELLIPSE FROM STEADY STATE SOLUTION

In the main text, we express the steady state solution of a single pillar in the array in terms of the complex amplitudes b_x and b_y. In this section, we determine the shape of the elliptical trajectory from this steady state solution.

First, we express the solution in terms of the physical displacement i.e. $x = \sqrt{2/\omega_x}\,\mathrm{Re}(b_x) \equiv A_x \cos(\Omega t - \phi_x)$, and likewise for y-displacement. In order to extract the shape of the elliptical trajectory, we look at the covariance matrix of the oscillatory motion, which is:

$$C = \begin{pmatrix} \langle x^2 \rangle & \langle xy \rangle \\ \langle xy \rangle & \langle y^2 \rangle \end{pmatrix} = \frac{1}{2} \begin{pmatrix} A_x^2 & A_x A_y \cos(\phi_x - \phi_y) \\ A_x A_y \cos(\phi_x - \phi_y) & A_y^2 \end{pmatrix}. \tag{12}$$

We could then calculate eigenvalues and eigenvectors of this oscillator-motion covariance matrix, to obtain the parameters of the ellipse A, θ, ζ (cf Figure 2 of the main text) as

$$A\cos|\zeta|, A\sin|\zeta| = \frac{A_x A_y}{\sqrt{2}} \left[\frac{1}{A_x^2} + \frac{1}{A_y^2} \pm \left(\left(\frac{1}{A_x^2} - \frac{1}{A_y^2} \right)^2 + \frac{4}{A_x^2 A_y^2} \cos^2(\phi_x - \phi_y) \right)^{1/2} \right]^{1/2}, \tag{13}$$

$$\theta = \arctan \left[\frac{A_x A_y}{2\cos(\phi_x - \phi_y)} \left(\frac{1}{A_y^2} - \frac{1}{A_x^2} + \left(\left(\frac{1}{A_x^2} - \frac{1}{A_y^2} \right)^2 + \frac{4}{A_x^2 A_y^2} \cos^2(\phi_x - \phi_y) \right)^{1/2} \right) \right], \tag{14}$$

$$\mathrm{sign}(\zeta) = \mathrm{sign}(x v_y - y v_x) = \mathrm{sign}(\sin(\phi_y - \phi_x)). \tag{15}$$

In the latter equation, the sense of rotation of the ellipse is determined by the sign of the ellipticity ζ, which can be determined from the sign of the angular momentum.

Supplementary Note 5. EFFECTIVE DISORDER MODEL

In the experimental array, the disorder primarily arises due to the variation of the pillar geometries in the fabrication process, which leads to a fluctuation of the isolated pillar frequencies (see Supplementary Note 7 for more details). Further, we assume that the disorder in the coupling strengths between the neighboring pillars is negligible in comparison to the on-site disorder. Therefore, it can be ignored in the disorder model. In this section, we explicitly write down the disordered on-site Hamiltonian, and visualise the physical interpretation of the different tight-binding model parameters (see Supplementary Figure 5).

As explained in the main text, the mean parameters of an isolated pillar i.e. normal mode frequencies $\omega \pm \Delta$ and orientation α are perturbed in the presence of disorder. It is convenient to describe this perturbation in the matrix

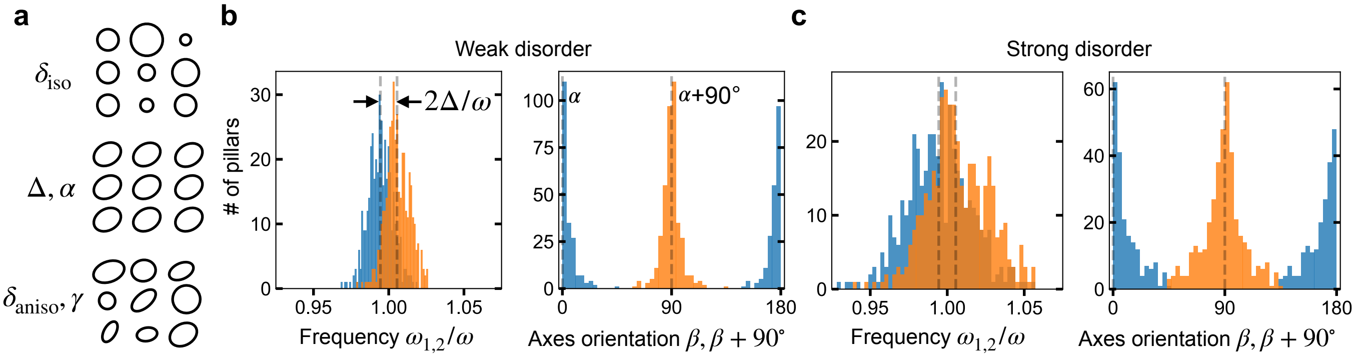

Supplementary Figure 5. **Effective disorder model.** **a**, Schematic visualisation of the three different terms of the disordered on-site Hamiltonian (Equation 16). The 3x3 array of ellipses represent the different pillars in the array. The shape of the ellipses represents the top-view of the pillar in the experiment, whereas the size signifies the varying height of the pillars. **b-c**, The distribution of uncoupled frequencies and orientations of all the pillars in the 20×20 array for weak (**b**) and strong (**c**) disorder levels. The weak (strong) disorder case refers to the tight-binding parameters that are used in Figure 5(**a**) (Figures 3 and 4) of the main text. Tight-binding parameter values for the case of strong disorder are specified in Supplementary Note 8 (see Supplementary Table I); for weak disorder, the parameters $\sigma_{\text{iso,aniso}}$ are reduced to 40% of their value.

notation of the 2x2 disordered on-site Hamiltonian, as shown below

$$H_{\mathrm{on-site}} = \begin{pmatrix} b_x^* & b_y^* \end{pmatrix} \left[\begin{pmatrix} \omega + \delta_{\mathrm{iso}} & 0 \\ 0 & \omega + \delta_{\mathrm{iso}} \end{pmatrix} + R(\alpha) \begin{pmatrix} -\Delta & 0 \\ 0 & \Delta \end{pmatrix} R^{-1}(\alpha) + R(\gamma) \begin{pmatrix} -\delta_{\mathrm{aniso}} & 0 \\ 0 & \delta_{\mathrm{aniso}} \end{pmatrix} R^{-1}(\gamma) \right] \begin{pmatrix} b_x \\ b_y \end{pmatrix}. \quad (16)$$

Here, $R(\alpha)$ and $R(\gamma)$ are the rotation matrices given by Equation 2. They are responsible for the couplings between the motion in x and y directions. Because of these terms, and the fact that α and γ are position-dependent, the dynamics of the array cannot be decoupled into independent scalar fields, and the treatment of mechanical polarization is essential.

The physical interpretation of the three different terms of Equation 16 is visualised in Supplementary Figure 5**a**. The disorder in the mean frequency ω (anisotropy parameter Δ) is simulated by the random variable $\delta_{\mathrm{iso}}(\delta_{\mathrm{aniso}})$, that is sampled from a gaussian distribution with mean zero and standard deviation σ_{iso} (σ_{aniso}). The fluctuation in the mean orientation α is characterised by the random angle parameter γ, which is sampled from a uniform distribution in the range $[0, 2\pi)$. For a particular choice of disorder parameters, the distribution of the uncoupled frequencies $\omega_{1,2}$ and the orientations β can be represented by a histogram plot as shown in Supplementary Figure 5**b,c**. Note that for reasons of simplicity, we consider that the isolated pillar parameters ω_1, ω_2 and β are uncorrelated among all the pillars in the array.

Supplementary Note 6. NEXT-TO-NEAREST NEIGHBOR COUPLING TERMS OF THE HAMILTONIAN

In this section, we show the explicit form of the next-to-nearest coupling term H_d of the tight-binding Hamiltonian.

The next-to-nearest coupling Hamiltonian H_d can be favourably written in terms of the complex amplitudes in the two diagonal directions $b_{\mathbf{r},\pm} = (b_{\mathbf{r},x} \pm b_{\mathbf{r},y})/\sqrt{2}$ ($\mathbf{r}$ is the position of the pillar), as shown below

$$H_d = -\underbrace{J_{\mathrm{d,ll}} \sum_{t,\langle\langle \mathbf{r},\mathbf{r}'\rangle\rangle_t} b_{\mathbf{r},t}^* b_{\mathbf{r}',t}}_{\text{n.n.n longitudinal coupling}} - \underbrace{J_{\mathrm{d,tt}} \sum_{t,\langle\langle \mathbf{r},\mathbf{r}'\rangle\rangle_{\bar{t}}} b_{\mathbf{r},t}^* b_{\mathbf{r}',t}}_{\text{n.n.n transversal coupling}}. \quad (17)$$

Here, $t = \{+, -\}$ labels the two diagonal directions, $\langle\langle \mathbf{r}, \mathbf{r}'\rangle\rangle_t$ indicates the next-to-nearest neighbor in the t direction. The bar symbol $\bar{t}$ in the transversal coupling term interchanges the two diagonal directions i.e. $\bar{+} = -$ and vice-versa.

Supplementary Note 7. CONTRIBUTIONS TO THE DISORDER IN THE EXPERIMENTAL DATA

The nanopillars in the array are nominally identical. However, despite the fact that the fabrication routines have been optimized to ensure accurate pattern transfer, a certain fabrication-induced disorder in the geometry of nanopillar resonators can not be avoided. In our case, this translates into a disorder in the pillars' resonance frequencies. In the nanopillar array under investigation, three types of disorder contribute to the overall disorder. To highlight the individual contribution of those mechanisms, this section presents an analysis of a different sample with a larger lattice constant, in which the coupling between neighboring pillars only plays a negligible role, facilitating the analysis of disorder. This sample has been fabricated in the same batch as the sample discussed in the main text.

- Random disorder between any two pillars in the center of an array: Slight fabricational differences in the radius, height or taper angle of the pillars can lead to variations of the eigenfrequencies of the two pillars. Optimization of the electron beam lithography and reactive ion etching allows to reduce the underlying artefacts. However, this disorder mechanism can not be fully eliminated and thus needs to be incorporated into the theoretical model. In Figure 6 this can be seen for each mode in the frequency distribution in the center of the array.

- Length gradient towards the edge of the array: This additional disorder mechanism is based on the diffusion limited reactive ion etching process, for which the etch rate is reduced in highly confined spaces. This essentially leads to a higher etch rate and hence longer pillars towards the edge of the array compared to the center of the array. As the surrounding of all pillars in the central area of the array can be considered effectively the same, systematic deviations are apparent only close to the edge of the array. This is also apparent from Figure 6. This systematic distortion is the reason, why we neglect the outermost pillars in all our experimental analyses and effectively only discuss a 16×16 rather than the full 20×20 array. In consequence, this systematic disorder mechanism is not included into the theoretical model.

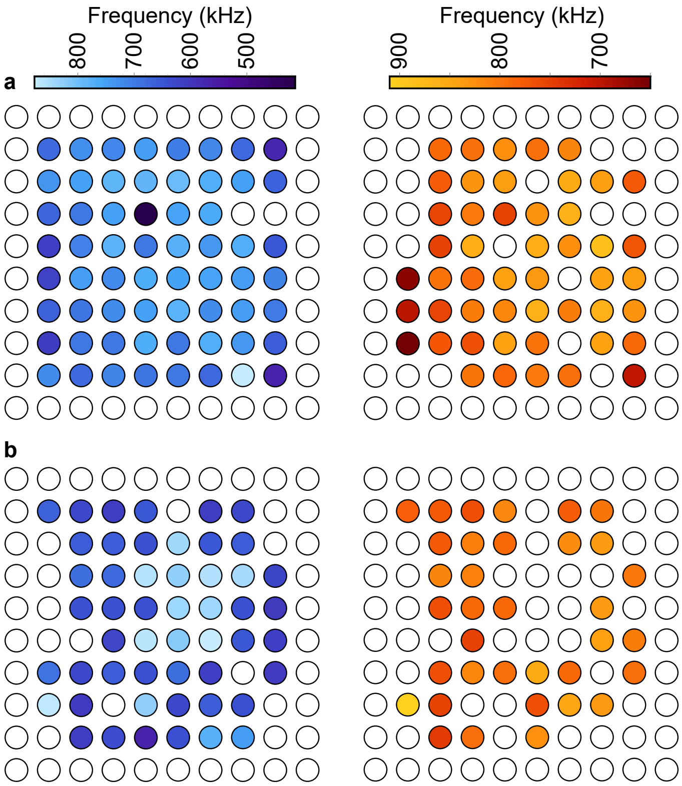

Supplementary Figure 6. **Experimental frequency disorder on a weakly coupled reference sample with lattice constant** $5\,\mu\mathrm{m}$**.** **a** and **b** depict the frequency distribution for the two main modes (blue and orange color scale, respectively) of two nominally identical arrays on the reference sample. As a result of the much larger lattice constant, the inter-pillar coupling on the reference sample is almost negligible. The different modes are shown on the left and the right, respectively, with the same color scale for the two identical arrays. Empty circles indicate pillars whose frequencies lie far from the two main modes.

- Anisotropy of the shape of an individual pillar: Although every pillar is nominally written as a circle in the electron-beam lithography process, typically there is some anisotropy to it so that the patterned shape is very slightly elliptical. Again, optimization of the electron beam lithography allows to reduce this effect to a minimal level. However, a certain, barely discernable ellipticity always remains. Typically we find that there is a different preferred direction and strength of this anisotropy randomly changing from sample to sample. This anisotropy appears as a considerable separation between the eigenfrequencies of the two orthogonal vibrational polarization directions in Figure 6, while the vibration direction within each mode is roughly the same for every pillar (more details in Supplementary Note 8). This disorder mechanism is also included in the theoretical model and introduced in Supplementary Note 5.

We note that while all three types of disorder contributing to the measurements discussed in the main text can be discerned in the reference sample and are apparent in Figure 6, the amount of disorder on the reference sample is not the same. More specifically, the reference sample turns out to exhibit a significantly higher anisotropy. Therefore, the quantitative disorder parameters obtained from the reference sample can not directly be employed in the analysis in the main text. See Supplementary Note 8 for more details on the determination of model parameters.

Supplementary Note 8. APPROXIMATE VALUES OF TIGHT-BINDING PARAMETERS FOR THE EXPERIMENTAL SYSTEM

As mentioned in the main text, it is impracticable to estimate the exact values of the tight-binding parameters. The major bottleneck behind estimating the isolated pillar parameters $(\omega, \Delta, \alpha, \delta_{\mathrm{iso}}, \delta_{\mathrm{aniso}})$ from the experimental data is that the trajectories are influenced due to the interaction with the neighbors. We came up with a solution to this conflict: to observe an array where the distance between pillars is sufficiently large that it is reasonable to ignore the couplings between the pillars (see Supplementary Note 7). For such a very weakly coupled array, we reliably estimate the isolated pillar frequencies and orientations, and analyze its spatial profile (see Supplementary Figure 6)

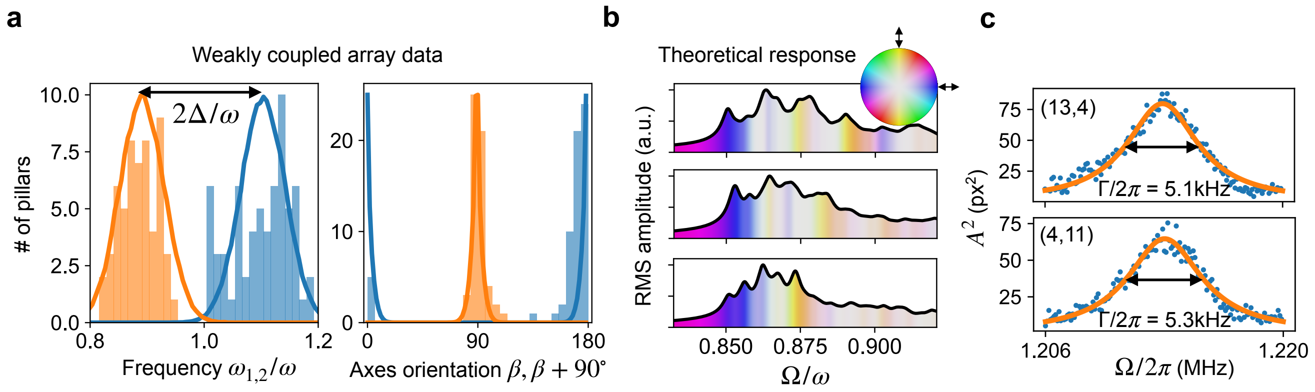

Supplementary Figure 7. **Determining approximate values of tight-binding parameters.** **a**, Experimentally obtained distribution of isolated pillar frequencies and orientations of a very weakly coupled array (different sample than in the main text). The isolated pillar parameters for this array are extracted by fitting this distribution (fit indicated with lines). **b**, Theoretically obtained response spectrums (with identical color scale as Figure 4 of the main text) for three different random realizations, but identical disorder parameters extracted from (a). **c**, Square of amplitude as a function of frequency for a single moving pillar mode. The FWHM of the fitted curve (orange) indicates the mechanical damping $\Gamma \approx 2\pi\times\ 5\,\mathrm{kHz}$.

**Parameter**	**Approximate value**	**Normalised value**
Mean frequency ω	$2\pi\times\ 1.33(2)\,\mathrm{MHz}$	1
Mean frequency anisotropy Δ	$2\pi\times\ 7.5(25)\,\mathrm{kHz}$	0.006
Mean orientation of smaller frequency mode β	$0(3)^\circ$	0°
Isotropic disorder σ_{iso}	$2\pi\times\ 27^{+22}_{-9}\,\mathrm{kHz}$	0.020
Anisotropic disorder σ_{aniso}	$2\pi\times\ 6.8(50)\,\mathrm{kHz}$	0.005
Longitudinal coupling (n.n) J_{ll}	$2\pi\times\ 30(25)\,\mathrm{kHz}$	0.022
Longitudinal coupling (n.n.n) $J_{\mathrm{d,ll}}$	$2\pi\times\ 15(13)\,\mathrm{kHz}$	0.011
Transversal coupling (n.n) J_{tt}	$2\pi\times\ 30(25)\,\mathrm{kHz}$	0.022
Transversal coupling (n.n.n) $J_{\mathrm{d,tt}}$	$2\pi\times\ 7.5(60)\,\mathrm{kHz}$	0.006
Mechanical damping Γ	$2\pi\times\ 5(1)\,\mathrm{kHz}$	0.004

Supplementary Table 1. **Estimated values of the tight-binding parameters for the experimental array in the main text.** The error in the approximate values (number in parentheses denotes the error such that the last digit of the error and quoted value correspond to each other) are estimated from either of the following: experimental data shown in the main text, the weakly coupled array in Supplementary Figure 7, the study of the coupling on pillar pairs in [1], comparing the experimental and simulated response spectrum. The normalised frequency parameter values are evaluated relative to the mean frequency ω.

and statistics (see Supplementary Figure 7**a**). We use this weakly coupled array data as a guide to estimate the approximate values of the isolated pillar parameters for the array used in the main text, cf. Supplementary Table I.

Below, we present the approximate values of the 10 tight-binding parameters for the array presented in the main text.

- Mean frequency ω: It is roughly equal to the mean of the two strongest resonances in the response spectrum (RMS amplitude vs. frequency) in Figure 4 of the main text i.e. $\omega \approx 2\pi\times 1.35\,\mathrm{MHz}$.

- Mean frequency anisotropy Δ: If it is too large $\Delta \gg \{\Gamma, J_{\mathrm{ll}}, J_{\mathrm{tt}}, J_{\mathrm{d,ll}}, J_{\mathrm{d,tt}}, \sigma_{\mathrm{iso}}, \sigma_{\mathrm{aniso}}\}$ (as is the case for the weakly coupled array), then the response spectrum would feature two different peaks corresponding to two orthogonal modes. If it is too small, then we would observe just a single band in the response spectrum. However, the experimentally observed response spectrum in the main text show none of the two cases, instead it exhibits resolved peaks with no clear sense of orientation within each peak. For $\Delta = 2\pi\times\ 7.55\,\mathrm{kHz}$ ($\Delta/\omega = 0.006$), we could qualitatively create this scenario in our theoretical model.

- Mean orientation of smaller frequency mode β: We have observed multiple arrays where frequency anisotropy Δ was large enough that we could associate orthogonal orientations to the two resonances in the response spectrum. For all such arrays, including the weakly coupled array data in Supplementary Figure 7**a**, the two

normal-mode orientations are close to 0° and 90°. For the experimental array in the main text, we observe that the trajectories are near horizontally polarized for smaller driving frequencies. Hence, we consider the smaller frequency mode to be at 0°.

- Disorder parameters σ_{iso} and σ_{aniso}: The theoretical model assumes that the disorder mainly arises in the isolated pillar parameters ω and Δ. For the weakly coupled array, we extract the disorder parameters as: $\sigma_{\text{iso}}/\omega = 0.036$ and $\sigma_{\text{aniso}}/\Delta = 0.155$, cf. Supplementary Figure 7**a**. However, the isotropic disorder appears to be too strong for the experimental array in the main text. This is because the response spectrum for these parameters exhibits a rough lineshape (see Supplementary Figure 7**b**), in contrast to the smooth peaks in the main text. Therefore, we take the approximate isotropic disorder as $\sigma_{\text{iso}}/\omega = 0.02$ (smaller than than that of uncoupled array). The diversity of the orientations in the two strongest resonances of the main text could be explained by selecting a larger anisotropic disorder $\sigma_{\text{aniso}}/\Delta = 0.907$ than the weakly coupled array. The distribution of the estimated isolated pillar frequencies and orientations for the array in the main text is shown in Supplementary Figure 5**c**.

- Coupling parameters $J_{\text{ll}}, J_{\text{d,ll}}, J_{\text{tt}}, J_{\text{d,tt}}$: Using the experimental study of the interaction strength as a function of distance between the pillars [1], we estimate that the coupling strengths are of order 10 kHz. We find that the theoretical model predicitions for $J_{\text{ll}} = J_{\text{tt}} = 30\,\text{kHz}$ agrees well with the experimental results. The diagonal neighbor coupling strengths are estimated to be smaller, cf. Supplementary Table I, because of the increased distance between the pillars.

- Mechanical damping Γ: In the frequency response, we look at the cases when only one (or maximum two) pillar is effectively moving. By fitting the individual pillar response spectrum with a Lorentzian for such cases, we estimate the mechanical damping as $\Gamma \approx 2\pi\times\ 5\,\text{kHz}$ (see Supplementary Figure 7**c**).

Supplementary Note 9. DETERMINATION OF THE LOCATION OF C POINTS IN THE STEADY STATE PATTERN

In Figure 5 of the main text, we show the topologically robust L lines and C points in the theoretically obtained steady state patterns, whereas only the C points in the experimentally obtained patterns. In this section, we describe the procedure of determining the locations of these polarization singularities [2], and convey that they are robust only for weak disorder levels.

In the first place, polarization singularities are defined for polarization fields in a continuous space, but here we consider the situation where the field is specified only on a discretized grid. However, the definition of polarization fields can be generalized also to this case in a consistent manner: If two neighboring grid points have opposite handedness (sgnζ), they must be separated by an L line. If a plaquette has a non-trivial winding number for the orientation θ, it must contain a C point. To determine smooth locations for the L lines and C points, we extend the field from the grid points to the entire plane by interpolation. The L lines can be obtained in the theoretical steady-state patterns as the zero contours of the ellipticity ζ, whereas C points are located at the nodes of the major-axis orientation θ, cf. Supplementary Figure 8**a**.

More concretely, we determine the location of the C points within the array as follows:

(i) First, we determine all the plaquettes where the C points are located. This can be done by considering the winding number of θ around a plaquette, which in the simplest case of a square lattice is

$$I = \frac{d(\theta_{\text{sw}}, \theta_{\text{se}}) + d(\theta_{\text{se}}, \theta_{\text{ne}}) + d(\theta_{\text{ne}}, \theta_{\text{nw}}) + d(\theta_{\text{nw}}, \theta_{\text{sw}})}{2\pi}, \tag{18}$$

with

$$d(\theta_1, \theta_2) = \theta_2 - \theta_1 + \pi\mathbb{Z} \in [-\pi/2, \pi/2). \tag{19}$$

The indices stand for north/south-east/west, respectively. This quantity I can be computed for a discrete grid, and its value being non-zero can be used to define a "C plaquette". Note that I by definition can only be an integer multiple of 1/2, since the differences of orientations $\theta_1 - \theta_2$ cancel around the loop and only the "modulo π" operation in the definition of each individual term d leads to a nonzero result. The sum of I over all plaquettes in an infinitely extended lattice is conserved (in a finite system, changes may come in from the boundaries, just as for vortices). We also mention in passing that in principle, if the orientation field θ on the lattice is originally obtained by evaluation of an underlying smooth field (defined in continuous space), the plaquettes with nonzero I need not contain the locations of the C points of this smooth field (they might e.g. sit in adjacent plaquettes, depending on the precise field configuration).

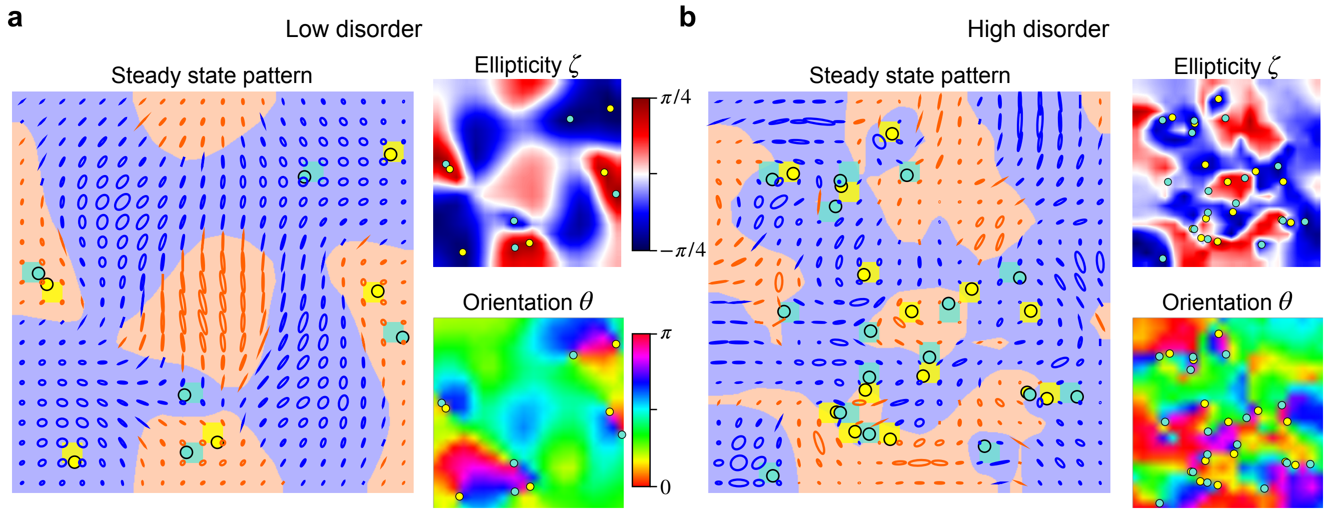

Supplementary Figure 8. Location of polarization singularities in theoretically obtained steady state pattern. The position of L lines and C points are determined in the steady state patterns for weak (**a**) and strong (**b**) disorder levels. The ellipticity is zero at L lines, which separates region of opposite handedness (blue and red regions). At the C points, the major-axis orientation θ has a singularity with winding index $I = \pm 1/2$ (indicated with different marker colors) and ζ has its extremum. For stronger disorder in **b**, the field pattern fluctuates rapidly within the array. Therefore, there are several singularities in the polarization field, and the L lines and C points can easily be destroyed by slightly varying the drive frequency. Tight-binding parameter values corresponding to the weak (strong) disorder are similar as in Supplementary Note 8, except the disorder parameters $\sigma_{\text{iso,aniso}}$ are decreased (increased) by 60% (100%).

(ii) In principle, we could simply indicate the plaquettes with nonzero I. However, we can use interpolation to obtain a smooth version of the orientation field, which then enables us to propose a more precise location of the C points (see Supplementary Figure 9). In order to identify the location of the C point within a plaquette, we use the fact that C points are mapped to the poles of the Poincaré sphere (when considering the mapping from the real-space plaquette to this sphere). Therefore, they are vortices of the field $\tau = \cos(2\zeta)e^{2i\theta}$ (real and imaginary parts of τ represent the projection of a point onto the equatorial plane of the Poincaré sphere). Hence, for each plaquette, we are interested in the solution of

$$\tau(x_0 + \delta x, y_0 + \delta y) = 0, \tag{20}$$

where (x_0, y_0) is the bottom-left corner of this plaquette. Using bilinear interpolation, we determine the value of τ at any arbitrary point inside the plaquette, and numerically determine the roots of the above equation, cf. Supplementary Figure 9. Note that it is possible to have multiple C points within the plaquette, subjecting to the condition that the sum of their winding index is equal to that of the winding index of the plaquette.

For weak disorder where the field is smooth, the polarization pattern in the vicinity of a C point is close to circular. One can then easily observe aspects such as how the annihilation of two C points influences the surrounding polarization field (see Supplementary Figure 10). For stronger disorder, the polarization pattern changes rapidly, with orientations of neighboring lattice points almost uncorrelated. Therefore, a larger fraction of the plaquettes are then classified as C plaquettes, and there are often pairs of directly neighboring plaquettes or even longer chains with C plaquettes of opposite winding index (see Supplementary Figure 8**b**). They can easily disappear (e.g. when sweeping the frequency) because there is always a closeby annihilation partner. The stronger the disorder gets, the more this behaviour is observed.

Supplementary Note 10. SYSTEMATIC TRENDS IN THE FREQUENCY EVOLUTION OF C POINTS IN THE OBSERVED PATTERNS

In Figure 5 of the main text, we display the evolution of the extracted C points in a small region of the array. In this section, we produce a more systematic study, where we track the number of extracted C points throughout the array as a function of the excitation frequency, around the largest resonance peak, cf. Supplementary Figure 11**a**. This analysis confirms that there are systematic trends; e.g. the density of C points depends on systematic aspects such as the wavelengths of the excited eigenmodes (see Supplementary Figure 11**b-e**). The C points form a cluster

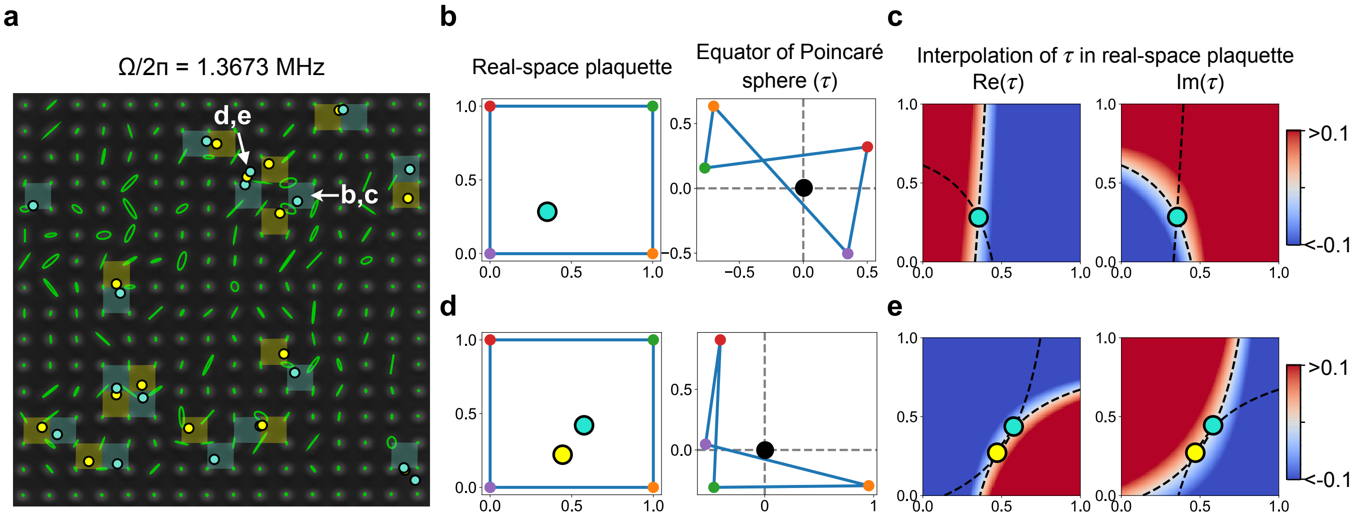

Supplementary Figure 9. Determining sub-lattice resolved location of C points in the steady-state pattern. **a**, Experimentally observed polarization pattern featuring C plaquettes ($I = \pm 1/2$ for yellow (+) and blue (-)) and proposed location of C points within the plaquettes. **b-c** and **d-e**, Demonstration of the algorithm with two example plaquettes (indicated in **a**) containing different number of C points. **b,d**, The four elliptical trajectories at the plaquette corners are mapped on to the equatorial plane of the Poincaré sphere (τ). The winding index of a plaquette is non-zero if the polygon constructed by joining the four mapped points encloses the C point (or the origin), indicated as a black circle. **c,e**, The position of the C point in the real-space plaquette is obtained by finding the roots of the interpolated function $\tau(x, y)$ (zero contour-levels of Re(τ) and Im(τ) are indicated with dashed lines).

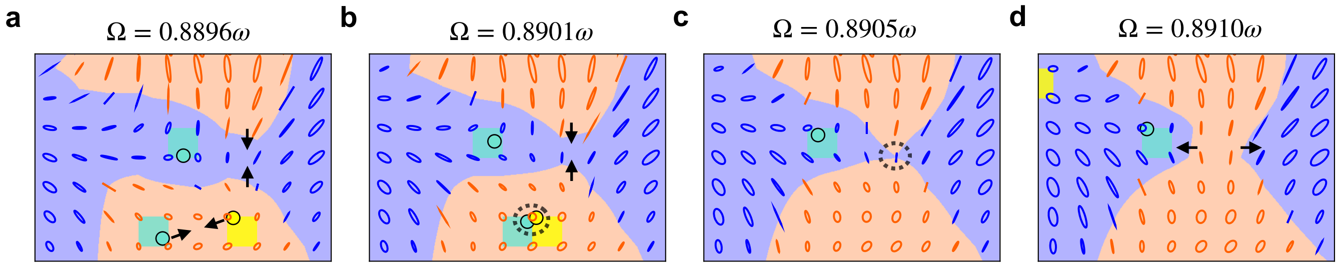

Supplementary Figure 10. Frequency evolution of topological singularities in the polarization patterns of slightly disordered arrays. Simulation of the motion of topological singularities as a function of frequency. **a-d**, Frequency evolution of the steady state pattern in a section of a slightly disordered array. The L line (C point) is robust, as it can only deform and move around with frequency (black arrows), unless it merges with another another L line (C point of opposite winding index) and split (annihilate), see the black-dashed circle in **c** (**b**).

near the short-wavelength regions of the array (e.g. near the edges of the array), whereas they are sparse near the long-wavelength regions (e.g. at the tip of the response spectrum Supplementary Figure 11**c**).

SUPPLEMENTARY REFERENCES

[1] J. Doster, S. Hoenl, H. Lorenz, P. Paulitschke, and E. M. Weig, “Collective dynamics of strain-coupled nanomechanical pillar resonators,” Nature Communications **10**, 5246 (2019).

[2] T. Fösel, V. Peano, and F. Marquardt, “L lines, C points and Chern numbers: understanding band structure topology using polarization fields,” New Journal of Physics **19**, 115013 (2017).

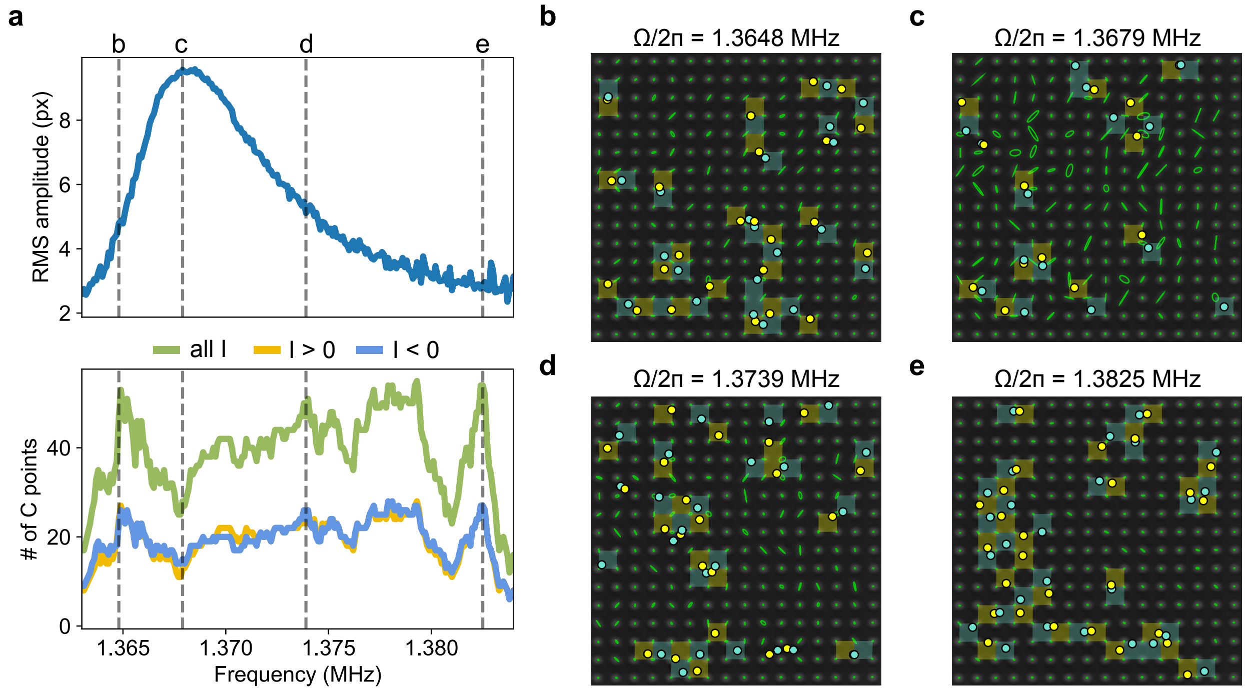

Supplementary Figure 11. Systematic trends in the frequency evolution of C points in the observed patterns. **a**, (top) RMS amplitude and (bottom) number of C points as a function of the excitation frequency around the largest resonance peak. **b-e**, Experimentally observed patterns with extracted C points at the frequencies indicated in (**a**).
